# Association of Migraine and Irritable Bowel Syndrome in Saudi Arabia: A Nationwide Survey

**DOI:** 10.1155/2022/8690562

**Published:** 2022-01-18

**Authors:** Khalid A. Bin Abdulrahman, Nawaf S. Alenazi, Saad B. Albishri, Faisal F. Alshehri

**Affiliations:** College of Medicine, Imam Mohammad Ibn Saud Islamic University (IMSIU), Riyadh, Saudi Arabia

## Abstract

Migraine is a primary headache disorder with a prevalence of 11.6% globally and 27% in Saudi Arabia. Irritable bowel syndrome (IBS) has a prevalence of 9.2% worldwide. The prevalence of IBS has not been established nationally. However, provincial studies for migraine and IBS have been conducted nationwide. There is a significant link between migraine and IBS globally. Migraineurs had a considerably greater prevalence of IBS than nonmigraineurs (OR = 2.49, 95% CI 2.22-2.78). Patients with IBS have 60% higher odds for migraines. This identifies an association that needs to be investigated in a nationwide manner. The study has two main aims. The first is to measure the prevalence of migraine and irritable bowel syndrome in Saudi Arabia. The second is to observe the association and the relationship between migraine and irritable bowel syndrome in Saudi Arabia. A cross-sectional study was conducted among the general population of Saudi Arabia between March 2021 and June 2021, whose ages are 15 years old or greater. Participants filled an online self-administered survey. The data collection tools included the Migraine Screen Questionnaire (MS-Q) for migraine symptoms, migraine severity (MIGSEV) scale for severity of migraine, and the IBS module of the Rome IV Diagnostic Questionnaire (R4DQ) for IBS symptoms and their subtype. With 2802 participants, the majority of the study samples were males, who constituted 52.5%. Among the study's sample, the prevalence of migraine consisted of 27.4%, and the prevalence of IBS was 16.4%. The odds of having IBS in migraineurs were much higher than in those without migraine (OR 4.127; 95% CI 3.325-5.121), and the association was statistically significant (*p* < 0.001). In conclusion, there is a strong association between migraine and irritable bowel syndrome in Saudi Arabia.

## 1. Introduction

Migraine is a common primary headache disorder with recurrent attacks lasting between 4 and 72 hours, causing moderate to severe unilateral throbbing pain, worsened by physical activity, and can be associated with nausea, photophobia, and phonophobia [1]. With a prevalence of 11.6%, migraine is the third most common disease in adults worldwide and the third leading cause of disability in those under 50 [2, 3]. In Saudi Arabia, migraineurs accounted for 27% of the population, with an adjusted 1-year prevalence of 25% [4, 5].

Four studies were conducted on the general public of different regions of Saudi Arabia, three to assess the prevalence of migraine headaches and a fourth study to assess migraine awareness. The first paper provides historical evidence of migraine prevalence. It was carried out in 1997 in Thuqbah, Saudi Arabia, using a modified version of the WHO criteria to screen for headaches in 23,227 participants. Their findings revealed that 5% of their participants suffered migraine headaches [6]. Nevertheless, the study population for this article was only the residents of the Thuqbah neighborhood in Khobar city. An article undertaken in the southern part of Saudi Arabia, Aseer, was aimed at determining the prevalence of migraine based on the ICHD-III criteria; they concluded that 12.3% of their sample had migraine headaches [7].

Furthermore, according to a study performed in Taif, located in the western part of Saudi Arabia, 78.5% of their sample experienced migraine headaches classified by the IHS migraine criteria [8]. However, this astronomical percentage of migraineurs is inconsistent with the rest of the study. In the final paper, nearly 40% of a study sample in Saudi Arabia's Eastern province were found to experience migraine headaches. However, the article directly asked if the participants had migraines without using any measuring tools [9]. A common disadvantage of these studies is that each one utilized a different methodology of evaluating migraine headaches, emphasizing the need for a large-scale study that presents migraine prevalence in various regions of Saudi Arabia using consistent criteria.

Irritable bowel syndrome (IBS) is a chronic idiopathic gastrointestinal condition characterized by abdominal pain that persists for more than one day per week in the previous three months and begins more than six months before diagnosis [10]. IBS can present as different subtypes, those with predominant constipation (IBS-C), diarrhea (IBS-D), mixed (IBS-M), or unsubtyped (IBS-U) [11]. IBS has long been known to impact people's quality of life severely. It is one of the most common gut-brain connection disorders, affecting approximately one out of ten people worldwide [12]. When evaluated using the Rome III criteria, the global prevalence of IBS was 9.2%, while it was 3.8% when assessed using the Rome IV criteria [13].

The prevalence of IBS has not been established nationally, with many cross-sectional studies only reporting the prevalence in a limited selection of the general populace. A study conducted in the central region investigated the prevalence of IBS using the Rome III criteria and found it to be 30.5% [14]. Additionally, two recent studies were conducted using the Rome IV criteria to measure the prevalence of IBS. The first was done in the southern region of Saudi Arabia, Jazan, which showed a total IBS prevalence of 16%. In contrast, the second study, conducted in Hail, located in the northern region of Saudi Arabia, discovered that 11.8% of participants had IBS [15, 16]. Overall, the literature on IBS prevalence in Saudi Arabia is higher than the worldwide estimates. Nonetheless, they did not reflect a national prevalence of IBS in the general population and resulted in greater variability in prevalence rates; thus, a nationwide prevalence investigation of IBS is required.

Both illnesses are diagnosed using symptom-based criteria. In terms of high prevalence, female preponderance, chronic and recurrent symptoms, pathogenesis, and burden to social and financial costs, migraine and IBS share many similar attributes [17]. Because various disorders have been related to the central nervous system and the enteric nervous system, the brain-gut axis is thought to substantially affect how neuronal disorders affect the GI tract [18]. Also, it is hypothesized that serotonin, central and visceral hypersensitivity, and hereditary factors are common pathogenesis pathways for migraine and IBS [17, 19].

Globally, various studies have shown a significant link between migraine and IBS. A cohort study that looked at the prevalence of migraine in IBS patients using data from a major US health plan discovered that those with IBS had a 60% higher risk of migraine than people without IBS (POR = 1.6, 95% CI 1.4–1.7) [20]. Additionally, a retrospective cohort based on Taiwan's National Health Insurance Research Database also observed that IBS incidence was nearly two times greater in the migraine cohort than in the comparator cohort [21]. Furthermore, according to a 2020 systematic review and meta-analysis, migraineurs had a considerably greater prevalence of IBS than nonmigraineurs (OR = 2.49, 95% CI 2.22-2.78) [22]. Nationally, the link has not been studied thoroughly; therefore, a large-scale nationwide study must fill this gap in the literature.

The study has two main aims. The first is to measure the prevalence of migraine and irritable bowel syndrome in Saudi Arabia. The second is to observe the association and the relationship between migraine and irritable bowel syndrome in Saudi Arabia. We hypothesize irritable bowel syndrome and migraine to have a direct connection, increasing the odds of having irritable bowel syndrome in migraine patients.

## 2. Materials and Methods

### 2.1. Study Design

This is an observational cross-sectional analytical study conducted in Saudi Arabia from March 2021 to June 2021. The study population consisted of Saudi Arabia's general population, aged 15 and older. Participants filled an online self-administered survey. The general population of Saudi Arabia is around 34.8 million. The target sample size was at least 384 based on 5% precision with a 95% confidence interval (CI). Data collectors were recruited from each of the five geographical regions of Saudi Arabia (Central, Eastern, Western, Northern, and Southern). Each data collector was asked to collect data by any means necessary, for example, through social media or by asking their acquaintances directly.

### 2.2. Data Collection Tools

The survey was divided into three sections. The first section focuses on the participants' demographic and personal information. The second section discusses migraine symptoms using the Migraine Screen Questionnaire (MS-Q) and their severity measured by the migraine severity (MIGSEV) scale. Irritable bowel syndrome manifestations are evaluated in the third section by utilizing the IBS module of the Rome IV Diagnostic Questionnaire (R4DQ).

The MS-Q is a five-question survey used in research and healthcare settings to test for migraines in the general population, developed based on the International Headache Society criteria. Each “Yes” on the questionnaire equals one point, whereas each “No” equals zero points. With a maximum score of 5, if the total score is equal to four or more, this indicates having migraine symptoms [23]. MS-Q has a sensitivity of 0.82 and a specificity of 0.97 [24]. Furthermore, the MIGSEV scale is a 4-item migraine severity questionnaire that categorizes people with low, moderate, or high overall migraine severity. MIGSEV is a reliable scale, with a Cronbach coefficient of 0.84 for physician evaluation and 0.86 for patient review [25].

The R4DQ was established based on the Rome IV diagnostic criteria and published in 2016 by the Rome Foundation [26]. In IBS, the questionnaire showed a sensitivity of 62.7% and specificity at 97.1% [10]. Six questions make up the IBS module of the questionnaire. When a participant answers “Once a week” or a greater frequency for the first question, “30%” or higher for the second to the fourth question, and “Yes” for the fifth question, they are labeled IBS-positive. The sixth question classifies participants into IBS subgroups.

### 2.3. Ethical Consideration

The study was approved by Imam Mohammad Ibn Saud Islamic University research ethics committee (project number 38-21; approval date, 16 March 2021). All writing is done in accordance with the ethical principles of the declaration of Helsinki. A brief description of the study was included with the survey link, with a full explanation on the survey's front page. Participants were told consent was given by filling the survey. Throughout the study, consent of all participants and data were gathered in complete confidence.

### 2.4. Statistical Analysis

For data analysis, IBM's SPSS v21 was used. Categorically measured variables were described using frequency and percentages. The normality assumption of the continuous variables was assessed using the histogram and the statistical Kolmogorov-Smirnov K-S test. In contrast, the equality of statistical variance assumption was evaluated using Levene's homogeneity of variance test. The correlations between categorically measured variables were evaluated using the chi-squared (*χ*^2^) test of independence. When the statistical assumptions of the chi-squared test expected counts were violated, a corrected likelihood ratio chi-squared test was utilized. A multivariate logistic regression model was used to analyze the significance of the participants' odds of migraine and IBS. The link between migraine and IBS odds and other important demographic factors in the general population was represented as an odds ratio with an accompanying 95% confidence interval. The alpha significance level was taken as 0.05.

## 3. Results

Two thousand eight hundred two participants submitted the survey, and all participants were eligible and accounted for in the analysis. 52.5% were males. The majority of replies (46.7%) came from people aged 20 to 29. Regarding marital status, 54.9% had never married, compared to 45.1% who had ever been married (married, divorced, or widowed). The percentage of the responses from different geographical regions in Saudi Arabia was close, with the majority from the western geographical area 23.7%. A complete listing of the samples' sociodemographic characteristics is presented in Table 1.

**Figure 1:**
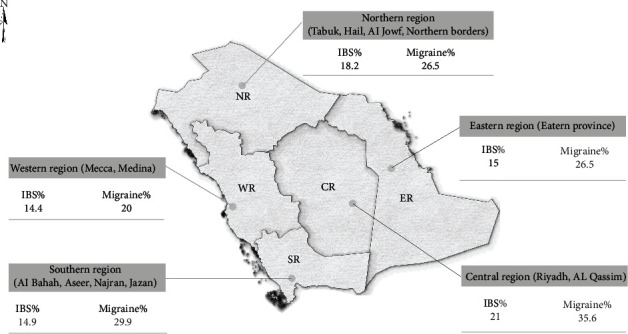
Prevalence of migraine and IBS in each geographical region of Saudi Arabia.

Seven hundred sixty-seven were found to be migraine-positive, showing a prevalence of 27.4%. Females had a considerably higher prevalence of migraine, with 37.5%, compared to 18.2% in males. 333 constituting the bulk of migraineurs (43.4%) experienced moderate migraine attacks, followed by 237 with mild migraine attacks (30.9%), and just 197 had severe migraine attacks (25.7%). IBS was less common than migraine in our sample, with 460 subjects fulfilling the Rome IV criteria for IBS, showing a prevalence of 16.4%. Females also had a higher percentage, with 20.9% compared to 12.4% for males.  Figure 1 depicts the prevalence of migraine and IBS in each geographical region of Saudi Arabia. To classify their IBS, subjects were asked to identify their stool form in the past three months, and the results revealed that the majority (34.8%) had IBS-M, followed by IBS-C (33%) and IBS-D (24.6%), and IBS-U was the least common (7.6%).

Females were shown to be significantly more likely to have both migraine and IBS in the bivariate analysis (*p* < 0.001), and in the multivariate regression analysis, the same result is seen for migraine and IBS, with males having 55.9% less probability of having migraine than females (OR 0.441; 95% CI 0.364-0.534; *p* < 0.001) and showing less chance of having IBS (OR 0.689; 95% CI 0.547-0.868; *p* = 0.002).

In the bivariate analysis, age groups differed in their chance of having a migraine. Individuals aged 40-49 years were shown to be substantially more likely than others to suffer from migraines than those aged 20-29 years, which were significantly less predisposed (*p* < 0.001). In IBS, age did not correlate significantly.

People residing in central Saudi Arabia were significantly more likely to have migraines (*p* < 0.001) and IBS (*p* = 0.013). Those living in the western region were considerably less likely to have migraines (*p* < 0.001). However, none of those relations is established in the multivariate regression analysis. Types of occupation converge significantly with migraines (*p* < 0.001); people working in education and freelance/charitable business and those unemployed/retired were more likely to have migraines. In comparison, those working in the military were significantly less inclined to migraines. In the multivariate binary regression analysis, people working in freelance jobs and charitable works were still significantly more inclined (2.22 times more) to be migraineurs than people in other employment or those unemployed (OR 2.223; 95% CI 1.339-3.693; *p* = 0.002). Students were found to be significantly less predicted (39.1% times less) for migraines compared to people who have other occupations (OR 0.609; 95% CI 0.467-0.794; *p* < 0.001).  Table 2 presents a detailed bivariate analysis of migraine and IBS with participants' sociodemographic characteristics, and an analysis of the association between migraine and IBS.

Participants with migraine were found to be significantly more likely to have IBS (*p* < 0.001); also, those with IBS showed a significant probability of having migraine (*p* < 0.001). This was also confirmed in our regression analysis, as those with migraine had higher odds (OR 4.127) of having IBS (95% CI 3.325-5.121; *p* < 0.001), in addition to those with IBS having an increased likelihood of having migraines (OR 3.304; 95% CI 2.632-4.147; *p* < 0.001). Migraineurs with severe symptoms were more likely to have IBS than migraineurs with moderate or lower migraine symptoms (*p* = 0.015). The analysis showed no correlation between IBS subtypes and migraine.

## 4. Discussion

This article is aimed at assessing the association between migraines and IBS in Saudi Arabia. The prevalence of migraine and IBS was 27.4% and 16.4%, respectively. In both conditions, females outnumbered males in terms of prevalence. The highest prevalence of migraine and IBS was seen in the central region. Both migraine and IBS were found to be substantially linked.

We found the prevalence of migraine to be similar to the prevalence of 27% reported in a previous study conducted in Saudi Arabia that used an independently developed questionnaire structured on the ICHD-III criteria [5]. This is notably higher than the worldwide prevalence [2]. Up to our knowledge, this is the first nationwide study conducted in the Kingdom of Saudi Arabia to assess IBS prevalence. Even though the prevalence of IBS reported in this study is considerably higher than the global prevalence reported in a meta-analysis of articles using the Rome IV criteria, this number is comparable to some worldwide reports [13, 27–29].

Various studies have been conducted to determine the prevalence of migraine and IBS in the general public. However, those studies were often conducted in Saudi Arabia's 13 provinces rather than geographical regions, making the comparison with our findings challenging. Additionally, different methodologies were utilized in the literature, complicating the comparison. A study conducted in Aseer, located in the southern part of Saudi Arabia, found the prevalence of migraine to be 12.3%, which is lower than the prevalence of migraine we measured in the southern region (29.9%) [7]. This could be due to other provinces in the southern part having a higher prevalence of migraine, increasing the prevalence for the whole geographical area. The southern region had a comparable IBS prevalence (14.9%) to a research conducted in Jazan (16%), which employed the same data collection tool for IBS symptoms measurement [15]. Using the same data collection method, a study in Hail city reported a lower prevalence of IBS (11.8%) than our findings in the northern region (18.2%) [16]. This could be because their study results were limited to Hail city, compared to our result, which pertains to the entirety of the northern region. Our report of IBS prevalence in the central region (21%) is lower than an article that utilized the old Rome II criteria, which found a prevalence of 30.5% [14]. This result is expected, as a systematic review showed that papers that used Rome III criteria scored a higher prevalence than those using Rome IV criteria [13].

Many causes have been hypothesized to impact the prevalence of migraine and IBS in Saudi Arabia. It is believed that inherited genetic variables play a significant role in migraine and IBS manifestation [30, 31]. This is particularly noteworthy given that consanguineous marriage is still common in some parts of the kingdom. Furthermore, a study conducted in Saudi Arabia found that lack of sleep, the use of certain medications, the consumption of caffeinated beverages, and working long hours all influence the occurrence of migraine [32]. Another study conducted in Saudi Arabia's northern region discovered that anxiety, depression, demanding professions, and excessive work hours are all attributed to IBS [33]. Further investigation is necessary to polish the factors influencing the high prevalence of migraine and IBS in Saudi Arabia.

In this study, females had a higher prevalence of migraine and IBS than males. This observation has been thoroughly established in the literature about Arab nations and globally [2, 13, 34, 35]. Females showed a higher prevalence of migraine and IBS, and the bivariate and regression analysis demonstrated that females had a higher probability of having migraine and IBS. A standard theory for the sex discrepancy in migraine and IBS is hormonal factors, especially sex hormones [36, 37]. Ultimately, more research is needed to investigate sex-related vulnerability to migraine headaches and IBS, including genetic and biological determinants and other environmental factors influencing migraine and IBS prevalence in females.

Migraine and IBS showed a strong association, as the presence of one disease increased the chance for the other. Migraineurs had 4.13 times the possibility of having IBS than those without migraine, which is higher than results shown by a recently published meta-analysis done on this subject (OR 2.49) [22]. The opposite is true, as IBS-positive participants had a higher risk of migraines. This link is also seen in the current literature [17, 20]. The cause of this relation is not yet fully understood. A plausible explanation could be defective serotonin, which has been linked to the pathophysiology of IBS and migraine since it modulates gut motility, secretion, and sensation and is an essential neurotransmitter in the central nervous system. This theory is supported by the similar management of both conditions, as 5-HT agonists and antagonists are beneficial in managing chronic hyperalgesic illnesses such as IBS, migraine, and fibromyalgia. Moreover, Cannabinoids have demonstrated dopamine blocking and anti-inflammatory properties in reducing trigeminovascular activity and improving gastrointestinal function, making them excellent for treating migraine, IBS, and fibromyalgia [17].

Diet has a significant impact on migraines and IBS, primarily via the brain-gut axis. Adhering to a low glycemic index diet, adequate fiber consumption, weight loss dietary regimens for overweight and obese patients, and supplementation with omega-3, vitamin D, and probiotics all have been shown to have positive effects on gut microbiota and the brain-gut axis [19, 31, 38]. Clinically, therapeutic elimination diets are useful, such as those that detect IgG antibodies to certain foods to identify triggers. There is strong evidence that an IgG-based elimination diet drastically improves the symptoms of both conditions, implying a relationship between the two via the enteric-nervous system mediated by serotonin and other inflammatory components [38, 39]. Diet has also been linked to the triggering of migraine and IBS symptoms, with studies finding food accounting for 26.9% and 84% of migraine and IBS triggers. Chocolate, dairy products, fruits, and legumes are all found to be common triggers of migraine and IBS. These foods are popular and regularly consumed in modern Saudi Arabia; hence, they could explain the severity of migraine and IBS symptoms [40, 41].

According to systematic review and meta-analysis, both disorders have a significant link to depression; this conclusion could be explained by the biopsychosocial model [17, 42, 43]. Additionally, we found migraineurs to have more severe migraine symptoms when comorbid with IBS. This finding may be comparable to a recent article reported that headache-related disability was more significant in participants with IBS symptoms than those without IBS, as measured by HIT-6 [44].

As hypothesized, migraineurs showed higher odds of having IBS than nonmigraineurs, showing a significant positive relationship between the two variables.

There are a few limitations to this paper that should be highlighted. For starters, no causal effects can be determined because the study is cross-sectional. Since this paper is based on the information given by respondents, there is a risk of recall bias. Even though migraine and IBS screening was done using validated questionnaires, the diagnosis was not confirmed clinically. Also, the study did not use the 13 provincial categorizations, and the five geographical regions were chosen based on ease of measurement and data comparability. The sensitivity and specificity of the MIGSEV scale have yet to be established to reflect validity. Moreover, the prevalence of IBS may vary depending on the criteria used, as evidenced by prior research [45–47].

## 5. Conclusions

There is evidence of a high prevalence of migraine headaches in the Saudi population (27.4%) and irritable bowel syndrome (16.4%). In terms of prevalence, females are much more likely than males to experience migraine and irritable bowel syndrome. Migraineurs who are IBS-positive are far more likely to have severe migraine symptoms than migraineurs without IBS. Our investigation observed a significant link between migraine and irritable bowel syndrome nationwide, confirming our hypothesis. These findings can be the basis to help create an impact on healthcare. National surveillance of migraine and IBS prevalence can bring awareness to the cost on the health and social care systems, mainly as both disorders have been attributed to a lower quality of life [17, 48, 49]. Clinically, a migraine patient may need to be evaluated for IBS and vice versa. In addition, measuring the severity of one disorder may aid in predicting the likelihood of developing the other. Nonetheless, more research is needed to determine the prevalence of migraine and IBS in Saudi Arabia's 13 provinces using consistent methodologies. In addition, while migraine is associated with IBS, there is no clear pathophysiological explanation for the correlation, and extensive research on overlapping factors in the migraine-IBS relationship is required to fill gaps in the literature.

## Figures and Tables

**Table 1:** Descriptive analysis of participants' sociodemographic characteristics.

*N* = 2802	*n* (%)
Sex	
Female	1332 (47.5)
Male	1470 (52.5)
Age group	
15-19 years	342 (12.2)
20-29 years	1308 (46.7)
30-39 years	431 (15.4)
40-49 years	503 (18)
50+ years	218 (7.8)
Marital state	
Never married	1538 (54.9)
Ever married	1264 (45.1)
Nationality	
Saudi	2715 (96.9)
Non-Saudi	87 (3.1)
Residence	
Central region	514 (18.3)
Eastern region	593 (21.2)
Northern region	412 (14.7)
Southern region	618 (22.1)
Western region	665 (23.7)
Occupation	
Unemployed/retired	501 (17.9)
Student	1147 (40.9)
Private sector	181 (6.5)
Military sector	88 (3.1)
Health sector	104 (3.7)
Governmental job	428 (15.3)
Education	274 (9.8)
Freelance job/charitable business	79 (2.8)

**Table 2:** Descriptive bivariate analysis of migraine and IBS with participants' sociodemographic characteristics and the association between migraine and IBS.

	Migraine		Test statistic	Irritable bowel syndrome		Test statistic
	No = 2035	Yes = 767	*p* value	No = 2342	Yes = 460	*p* value
Sex						
Female	832 (40.9)	500 (65.2)	*χ* ^2^ (1) = 131.94	1054 (45)	278 (60.4)	*χ* ^2^ (1) = 36.7
Male	1203 (59.1)	267 (34.8)	*p* < 0.001	1288 (55)	182 (39.6)	*p* < 0.001
Age group						
15-19 years	253 (12.4)	89 (11.6)	*χ* ^2^ (4) = 36.99	291 (12.4)	51 (11.1)	*χ* ^2^ (4) = 2.034
20-29 years	1011 (49.7)	297 (38.7)	*p* < 0.001	1100 (47)	208 (45.2)	*p* = 0.729
30-39 years	300 (14.7)	131 (17.1)		359 (15.3)	72 (15.7)	
40-49 years	323 (15.9)	180 (23.5)		412 (17.6)	91 (19.8)	
50+ years	148 (7.3)	70 (9.1)		180 (7.7)	38 (8.3)	
Marital state						
Never married	1183 (58.1)	355 (46.3)	*χ* ^2^ (1) = 31.58	1289 (55)	249 (54.1)	*χ* ^2^ (1) = 0.13
Ever married	852 (41.9)	412 (53.7)	*p* < 0.001	1053 (45)	211 (45.9)	*p* = 0.72
Nationality						
Non-Saudi	54 (2.7)	33 (4.30)	*χ* ^2^ (1) = 5.03	73 (3.1)	14 (3)	*χ* ^2^ (1) = 0.007
Saudi	1981 (97.3)	734 (95.7)	*p* = 0.025	2269 (96.9)	446 (97)	*p* = 0.934
Residence						
Central region	331 (16.3)	183 (23.9)	*χ* ^2^ (1) = 38.15	406 (17.3)	108 (23.5)	*χ* ^2^ (1) = 12.70
Eastern region	436 (21.4)	157 (20.5)	*p* < 0.001	504 (21.5)	89 (19.3)	*p* = 0.013
Northern region	303 (14.9)	109 (14.2)		337 (14.4)	75 (16.3)	
Southern region	433 (21.3)	185 (24.1)		526 (22.5)	92 (20)	
Western region	532 (26.1)	133 (17.3)		569 (24.3)	96 (20.9)	
Occupation type						
Unemployed/retired	329 (16.2)	172 (22.4)	*χ* ^2^ (7) = 58.54	415 (17.7)	86 (18.7)	*χ* ^2^ (7) = 9.19
Student	902 (44.3)	245 (31.9)	*p* < 0.001	956 (40.8)	191 (41.5)	*p* = 0.239
Private sector	139 (6.8)	42 (5.5)		165 (7)	16 (3.5)	
Military sector	72 (3.5)	16 (2.1)		73 (3.1)	15 (3.3)	
Health sector	74 (3.6)	30 (3.9)		85 (3.6)	19 (4.1)	
Governmental job	295 (14.5)	133 (17.3)		356 (15.2)	72 (15.7)	
Education	179 (8.8)	95 (12.4)		224 (9.6)	50 (10.9)	
Freelance job/charitable business	45 (2.2)	34 (4.4)		68 (2.9)	11 (2.4)	
Irritable bowel syndrome (IBS)						
Negative	1829 (89.9)	513 (66.9)	*χ* ^2^ (1) = 214.63	—	—	
Positive	206 (10.1)	254 (33.1)	*p* < 0.001	—	—	

## Data Availability

If deemed suitable, the data reported in this study are available upon request from the corresponding author.
